# Is Invagination Anastomosis More Effective in Reducing Clinically Relevant Pancreatic Fistula for Soft Pancreas After Pancreaticoduodenectomy Under Novel Fistula Criteria: A Systematic Review and Meta-Analysis

**DOI:** 10.3389/fonc.2020.01637

**Published:** 2020-08-21

**Authors:** Zhe Cao, Wenhao Luo, Jiangdong Qiu, Yueze Liu, Lianfang Zheng, Taiping Zhang

**Affiliations:** ^1^Department of General Surgery, Peking Union Medical College Hospital, Chinese Academy of Medical Sciences and Peking Union Medical College, Beijing, China; ^2^Department of Nuclear Medicine, Peking Union Medical College Hospital, Chinese Academy of Medical Sciences and Peking Union Medical College, Beijing, China; ^3^Clinical Immunology Center, Chinese Academy of Medical Sciences and Peking Union Medical College, Beijing, China

**Keywords:** duct-to-mucosa, surgery, invagination, pancreatic fistula, soft pancreas, pancreatic texture, new criteria

## Abstract

**Objective:** To define the effectiveness of different anastomosis on clinically relevant postoperative fistula in patients with soft pancreas using the newest version of the fistula definition and criteria.

**Background:** Different criteria of clinically relevant postoperative pancreatic fistula (POPF) result in the optimal anastomosis technique remaining controversial.

**Methods:** PubMed, Embase, Web of Science, the Cochrane Central Library, and ClinicalTrials.gov were systematically searched up to 20 April 2020, and were evaluated by Preferred Reporting Items for Systematic Reviews and Meta-analysis (PRISMA) guidelines. Randomized controlled trials comparing duct-to-mucosa anastomosis vs. invagination anastomosis in pancreatic surgery were included.

**Result:** Seven studies involving 1,110 participants were included. Using the postoperative pancreatic fistula definition provided by the International Study Group of Pancreatic Surgery 2016, the incidence rate of grade B/C pancreatic fistula was significantly lower in patients experiencing invagination anastomosis than in those undergoing duct-to-mucosa anastomosis. Four of seven trials comparing invagination with duct-to-mucosa anastomosis in patients with a soft pancreas showed that invagination was significantly better than duct-to-mucosa anastomosis in controlling pancreatic fistula formation, but no significant difference was detected between the two anastomosis techniques in patients with a hard pancreas. No significant difference in the length of hospital stay or postoperative mortality rate was found between the two methods.

**Conclusion:** This study demonstrated superiority of invagination anastomosis over duct-to-mucosa anastomosis in reducing the risk of Grade B/C postoperative pancreatic fistula using the ISGPS 2016 definition, but it does not significantly reduce the mortality rate or length of hospital stay. The effect of invagination in reducing pancreatic fistula formation is obvious in patients with a soft pancreas, but there is no significant difference between the two anastomosis techniques in patients with a hard pancreas. We found a lower rate of clinically relevant postoperative pancreatic fistula in the invagination group, in patients with a soft pancreas.

## Background

Pancreaticoduodenectomy (PD) is one of the most complex and difficult procedures among all surgeries. PD is the most common treatment method for patients with a resectable tumor of the pancreatic head and periampullary region or a suspicious mass or nodule in that area with obvious clinical manifestations. The use of more anastomoses in abdominal surgery increases the risk of developing a postoperative fistula, which may result in various postoperative complications. A postoperative pancreatic fistula (POPF) remains one of the most common complications of PD and can lead to abdominal infection, bleeding, and sepsis, all of which lead to a longer postoperative hospital stay, severe and potentially fatal complications, and higher mortality (1).

Pancreaticojejunostomy and pancreaticogastrostomy are two common methods for digestive reconstruction after PD. Duct-to-mucosa anastomosis and invagination anastomosis are the two major pancreaticojejunostomy techniques, which can also be applied to pancreaticogastrostomy.

Several recent studies have compared the efficacy of invagination and duct-to-mucosa techniques in preventing POPF, but with conflicting results (2–4). Notably, Hua et al. (2). used the definition suggested by the International Study Group of Pancreatic Surgery (ISGPS) in 2005 and found that invagination is not better than duct-to-mucosa anastomosis in terms of reducing POPF but reduce Grade B/C POPF. Sun et al. (3) found no significant difference between invagination and duct-to-mucosa anastomosis; however, they also used the POPF definition proposed by the ISGPS 2005. Ratnayake et al. (5) conducted a network meta-analysis to prove that duct-to-mucosa pancreaticogastrostomy can reduce the rate of POPF, but the ISGPS 2005 definition was still employed. The ISGPS 2005 definition is problematic because clinically non-relevant pancreatic fistulas are not distinguished from total pancreatic fistulas. Therefore, the latest POPF definition released by the ISGPS in 2016 excludes grade A POPF and redefines it as a biochemical fistula, which is more suitable in guiding clinical treatments as well as for meeting clinical needs.

In this study, we used the POPF definition proposed by the ISGPS in 2016 to compare invagination with duct-to-mucosa anastomosis for clinically relevant POPF only (6).

The pancreatic fistula risk score (FRS) is used to predict the risk of a pancreatic fistula after PD. According to the main risk factors for POPF, the FRS summarizes four main indicators: the texture of the pancreas, pathology, diameter of the main pancreatic duct, and intraoperative blood loss. The alternative FRS for PD (a-FRS) is based on three intraoperatively available variables: duct size, pancreatic texture, and body mass index (7). According to both the FRS and a-FRS, patients with a soft pancreas are more likely to develop POPF, but there is a lack of systematic reviews to support this (8). Moreover, few studies have systematically and comprehensively compared the efficacy of the two techniques in preventing clinically relevant POPF or POPF in patients with a soft pancreas.

Furthermore, we also compared invagination with duct-to-mucosa anastomosis in patients with a soft pancreas and hard pancreas, respectively. Finally, we compared the mortality rate and length of hospital stay after PD between patients who underwent duct-to-mucosa and invagination anastomosis.

## Materials and Methods

This systematic review was established by the guidelines of Preferred Reporting Items for Systematic Reviews and Meta-analysis (PRISMA) statement (9).

### Study Selection

Relevant studies were searched and identified by individually searching the following databases: PubMed, Embase, Web of Science, the Cochrane Central Library, and ClinicalTrials.gov up to 20 April 2020. For all databases, the search strategy involved use of the following key terms: “duct-to-mucosa” “pancreatic fistula” “pancreaticoduodenectomy” and “invagination.” The search was limited to publications of randomized controlled trials (RCTs). This meta-analysis adhered to the Critical Appraisal Skills Programme Checklist. An eligibility assessment was performed by two independent reviewers (WL and ZC). Disagreements between reviewers were resolved by group discussions and consensus.

### Inclusion and Exclusion Criteria

Eligibility was assessed by two independent reviewers (WL and ZC), with consensus reached by discussing conflicts with a third investigator (TZ). Assessments were performed and repeated twice. First, the titles and abstracts were assessed. The full text of potentially qualified studies was then obtained and assessed. No reviewers were blinded to the authorship of the studies. Dissertations, conference proceedings, and in non-English studies were excluded. There were no restrictions on the history of pancreatic diseases, the follow-up duration, or the reoperation times. To evaluate the effects of reducing pancreatic fistula of invagination and duct-to-mucosa anastomosis, we included those relevant studies.

### Outcomes of Interest

The primary outcome measure was the rate of clinically relevant POPF (grades B and C). We use the newest definition of POPF formulated by the ISGPS. The secondary outcome measures were the mortality rate and length of hospital stay. In the subgroup analysis, we compared the rate of clinically relevant POPF between the two anastomosis techniques in patients with a soft pancreas and hard pancreas, respectively.

### Data Collection

The data extracted included: first author, year of study, country of origin, number of patients, population characteristics, and POPF rate. The data were extracted and cross-checked independently by two authors (WL and ZC). Disagreements were resolved through discussions with a third reviewer (TZ) until a consensus was reached.

### Evaluation of Quality of Evidence

The methodological quality of the selected studies was blindly evaluated by two independent reviewers (WL and ZC). Disagreements were discussed among the group and resolved by a third assessor (TZ). Quality was assessed using the CASP Checklist, which evaluates the risk of bias and comprises 11 items related to methodological quality and statistical reporting. Discrepancies and disagreements were resolved by consensus.

### Statistical Analysis

Data were entered into the Cochrane Collaboration's Review Manager program (RevMan version 5.3; Cochrane Collaboration, Oxford, UK). We analyzed the standardized mean differences with 95% confidence intervals (CIs) and performed tests of heterogeneity (*I*^2^) for outcomes. Fixed-effects or random-effects models were used accordingly. Subgroup analysis stratified by the effect of pancreatic texture on reducing POPF was performed. Funnel plots were used to detect possible publication bias based on the primary outcome (incidence rate of POPF) and secondary outcomes (mortality and length of hospital stay).

## Results

### Literature Search

**Figure 1:**
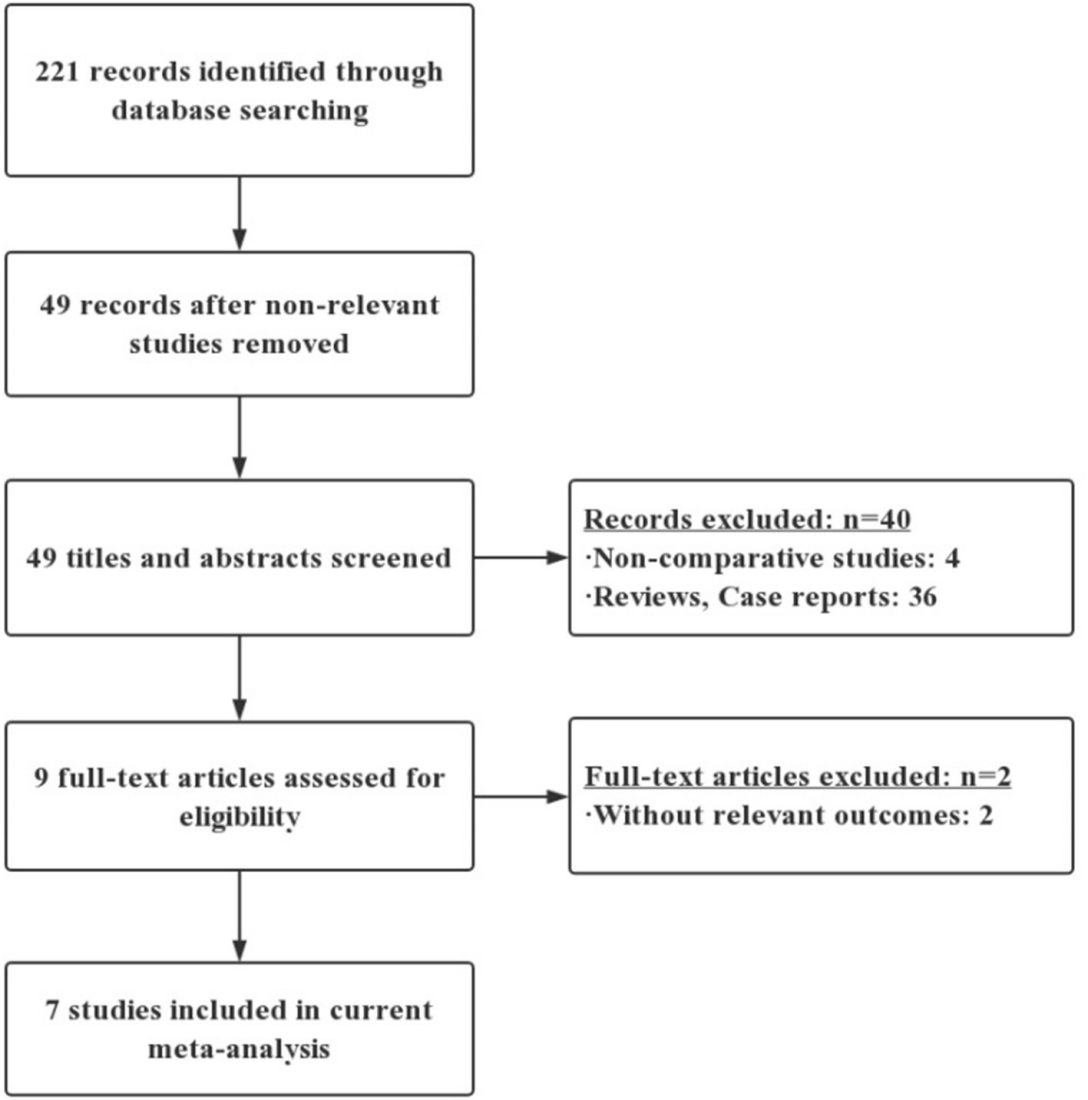
Inclusion and exclusion criteria.

A flow diagram of the literature search is shown in Figure 1. Among 221 unique articles, nine fulfilled the inclusion criteria. Initially, through the electronic database search, we identified 221 citations. Examinations of the reference lists in all relevant papers, recent editorials, and related review articles yielded no further studies for evaluation. Non-RCTs were excluded, leaving 49 citations. Nine articles were then selected after reading the titles and abstracts. After careful reading of these nine full-text articles, two studies were excluded because they did not include POPF outcomes. The remaining seven RCTs were included in the qualitative analysis and the final meta-analysis (10–16).

### Study Characteristics

The characteristics of each RCT are presented in Table 1. Our meta-analysis included 1,110 patients (640 men, 470 women) who underwent PD in five different countries. Duct-to-mucosa anastomosis was performed in 557 patients, and invagination anastomosis was performed in 553. The results of the quality assessment of the included RCTs are shown in Table 2.

**Table 1:** Characteristics of the included studies.

**References**	**Country**	**Year**	**Design**	**Age, mean (range or** ***SD*****)**		**No. of patients (M/F)**		**Texture of pancreas (Soft/hard)**	
				**D-to-M**	**Inv**	**D-to-M**	**Inv**	**D-to-M**	**Inv**
Senda et al. (15)	Japan	2017	RCT	66 (36–84)	68 (22–81)	61 (36:25)	59 (36:23)	31/30	30/29
Singh et al. (16)	India	2018	RCT	53.4 (12.1)	51.5 (14.2)	97 (63:34)	96 (63:33)	42/55	48/48
Maemura et al. (11)	Japan	2015	RCT	64 (49–79)	66 (45–85)	32 (19:13)	21 (14:7)	25/7	16/5
Xu et al. (12)	China	2015	RCT	58.17 ± 11.72	58.19 ± 10.70	153 (82:71)	155 (84:71)	95/58	104/51
Bai et al. (14)	China	2015	RCT	62 (32–79)	64 (21–78)	64 (38:26)	68 (39:29)	36/28	44/24
El Nakeeb et al. (13)	Egypt	2015	RCT	54 (12–73)	54 (20–75)	53 (34:19)	54 (33:21)	25/28	27/27
Berger et al. (10)	America	2009	RCT	68 (32–84)	68 (41–90)	97 (45:52)	100 (54:46)	50/47	51/49

**Table 2:** The quality assessment of the included RCTs.

**References**	**1**	**2**	**3**	**4**	**5**	**6**	**7**	**8**	**9**	**10**	**11**
Senda et al. (15)	1	1	1	1	1	1	1	1	1	1	1
Singh et al. (16)	1	1	1	0.5	1	1	1	1	1	1	1
Maemura et al. (11)	1	0.5	1	0	1	1	1	1	0.5	1	1
Xu et al. (12)	1	1	1	1	1	1	1	1	1	1	1
Bai et al. (14)	1	1	0.5	0.5	1	0	1	1	0.5	1	1
El Nakeeb et al. (13)	1	1	1	0.5	1	1	1	1	0.5	1	1
Berger et al. (10)	1	1	1	1	1	1	1	1	1	1	1

### Primary Outcome

#### POPF

**Figure 2:**
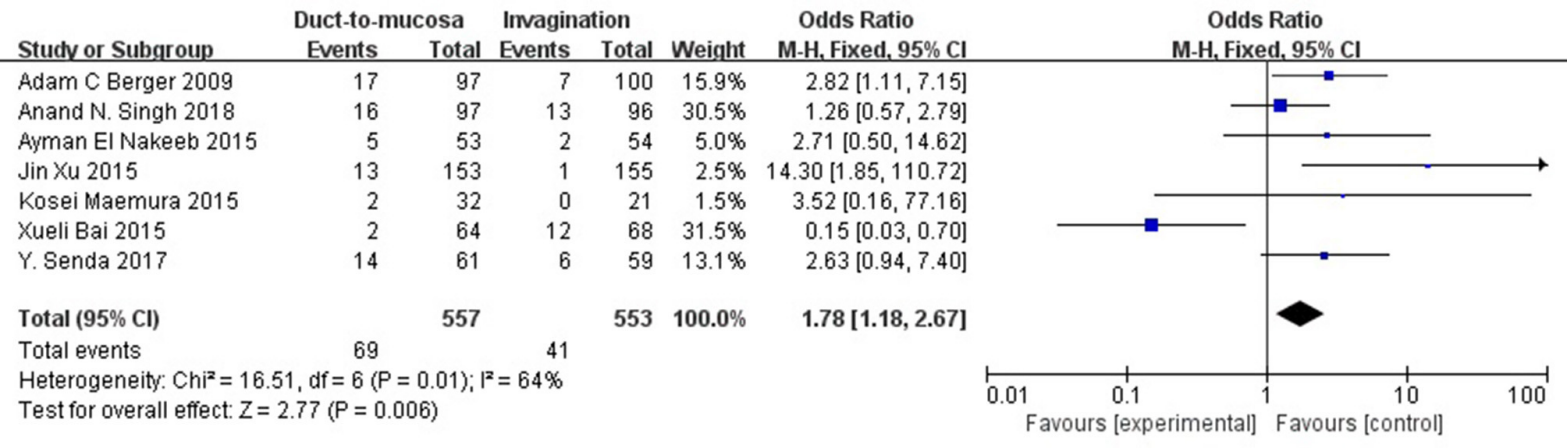
The comparison of incidence of POPF (grade B/C) between patients undergoing invagination and duct-to-mucosa. Incidence of clinically relevant POPF (grade B/C) was lower in patients undergoing invagination than in those undergoing duct-to-mucosa anastomosis (odds ratio [OR] = 1.78, 95% CI = 1.18–2.67, *P* = 0.006).

All of the included studies reported the POPF rate. The POPF rate was 12.4% (69/557) in the duct-to-mucosa group and 7.4% (41/329) in the invagination group. The incidence of Grade B/C POPF was lower in patients experiencing invagination than in those duct-to-mucosa anastomosis (odds ratio [OR] = 1.78, 95% CI = 1.18–2.67, *P* = 0.006) (Figure 2).

### Secondary Outcomes

#### Length of Hospital Stay

**Figure 3:**
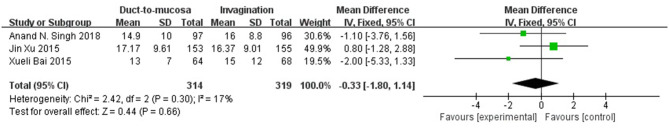
Three RCTs reported the length of hospital stay with useful data for analysis. No significant difference was found between the duct-to-mucosa and invagination techniques. No significant heterogeneity was present (Q statistic = 2.42, *P* = 0.30, *I*^2^ = 17%). The fixed-effects model was indicated; again, no significant difference was observed between the two groups (relative risk = −0.33, 95% CI = −1.80–1.14, *P* = 0.66).

Three RCTs reported the length of hospital. No significant difference was found between the two techniques. No significant heterogeneity was present (Q statistic = 2.42, *P* = 0.30, *I*^2^ = 17%). Data were analyzed using the fixed-effects model, which showed no significant heterogeneity (relative risk = −0.33, 95% CI = −1.80–1.14, *P* = 0.66) (Figure 3).

### Overall Mortality

**Figure 4:**
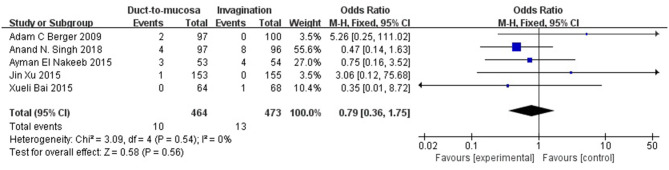
The postoperative mortality rates were reported in five RCTs. The overall mortality rate was 2.45% (2.16% in the duct-to-mucosa group and 2.75% in the invagination group). Data were analyzed using the fixed-effects model, which showed no significant heterogeneity (Q statistic = 3.09, *P* = 0.54, *I*^2^ = 0%). No significant difference was observed between the two groups (OR = 0.79, 95% CI = 0.36–1.75, *P* = 0.56).

The mortality rates were reported in five RCTs. The overall mortality rate was 2.45% (2.16% in the duct-to-mucosa group and 2.75% in the invagination group). The fixed-effects model was indicated but no significant difference was observed between the two groups (Q statistic = 3.09, *P* = 0.54, *I*^2^ = 0%). No significant difference was observed between the two groups (OR = 0.79, 95% CI = 0.36–1.75, *P* = 0.56) (Figure 4).

### Subgroup Analysis

**Figure 5:**
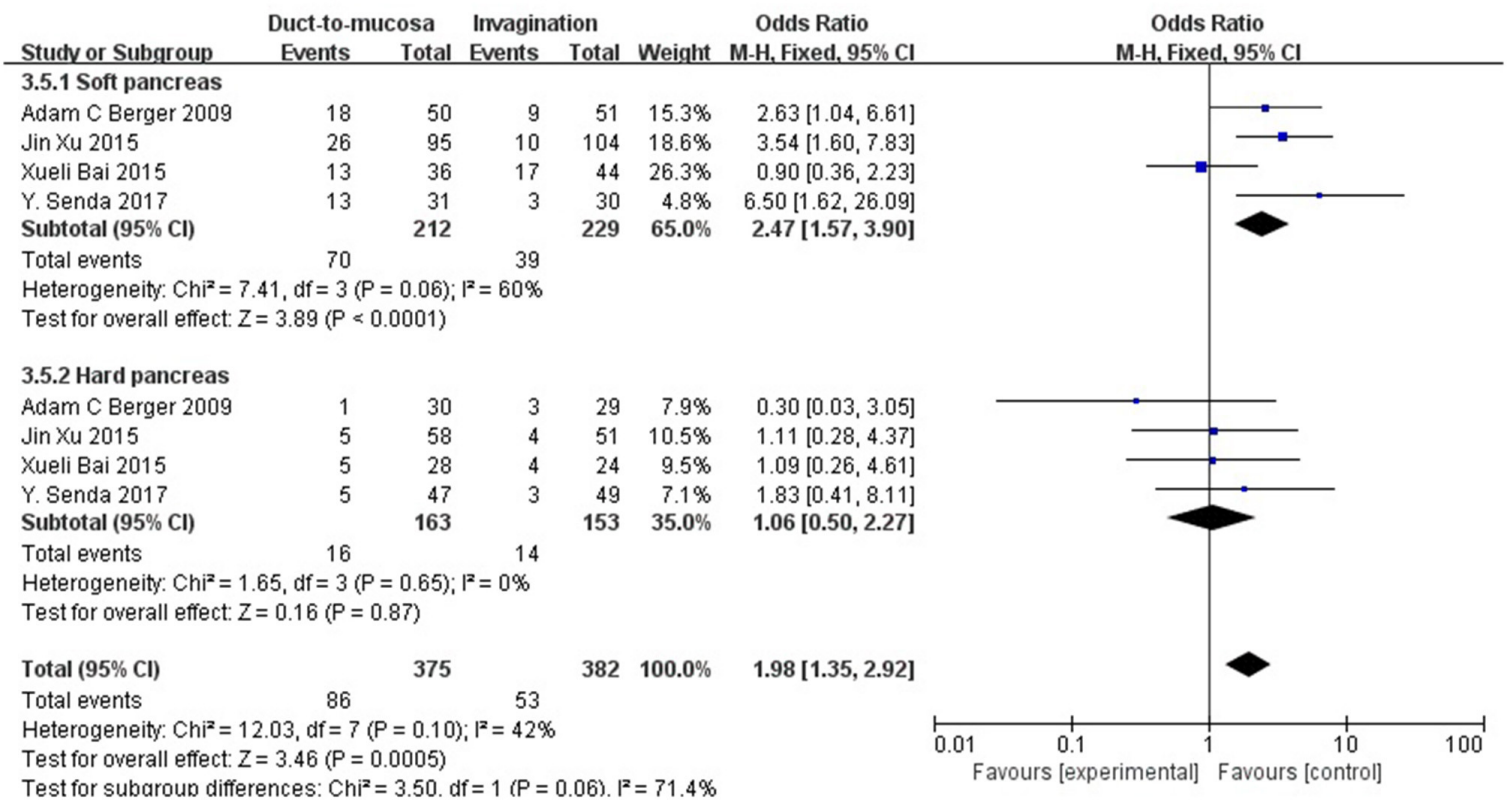
A subgroup analysis for patients with a soft pancreas. These four trials comparing invagination with duct-to-mucosa anastomosis in patients with a soft pancreas showed that invagination is much better than duct-to-mucosa anastomosis in controlling pancreatic fistula (OR = 2.47, 95% CI = 1.57–3.90, *P* < 0.0001). Four trials comparing invagination with duct-to-mucosa anastomosis in patients with a hard pancreas showed no significant difference between the two techniques in controlling pancreatic fistula (OR = 1.06, 95% CI = 0.50–2.27, *P* = 0.87).

Considering that soft pancreatic texture may be a significant risk factor for POPF, a subgroup analysis of soft pancreas patients was performed. Four studies involving 441 patients with a soft pancreas were pooled and analyzed. These four trials, comparing invagination with duct-to-mucosa anastomosis in patients with a soft pancreas, showed that invagination is much better than duct-to-mucosa anastomosis in controlling pancreatic fistula (OR = 2.47, 95% CI = 1.57–3.90, *P* < 0.0001). Four trials comparing invagination with duct-to-mucosa anastomosis in patients with a hard pancreas showed no significant difference between the two techniques in controlling pancreatic fistula (OR = 1.06, 95% CI = 0.50–2.27, *P* = 0.87) (Figure 5).

### Publication Bias

**Figure 6:**
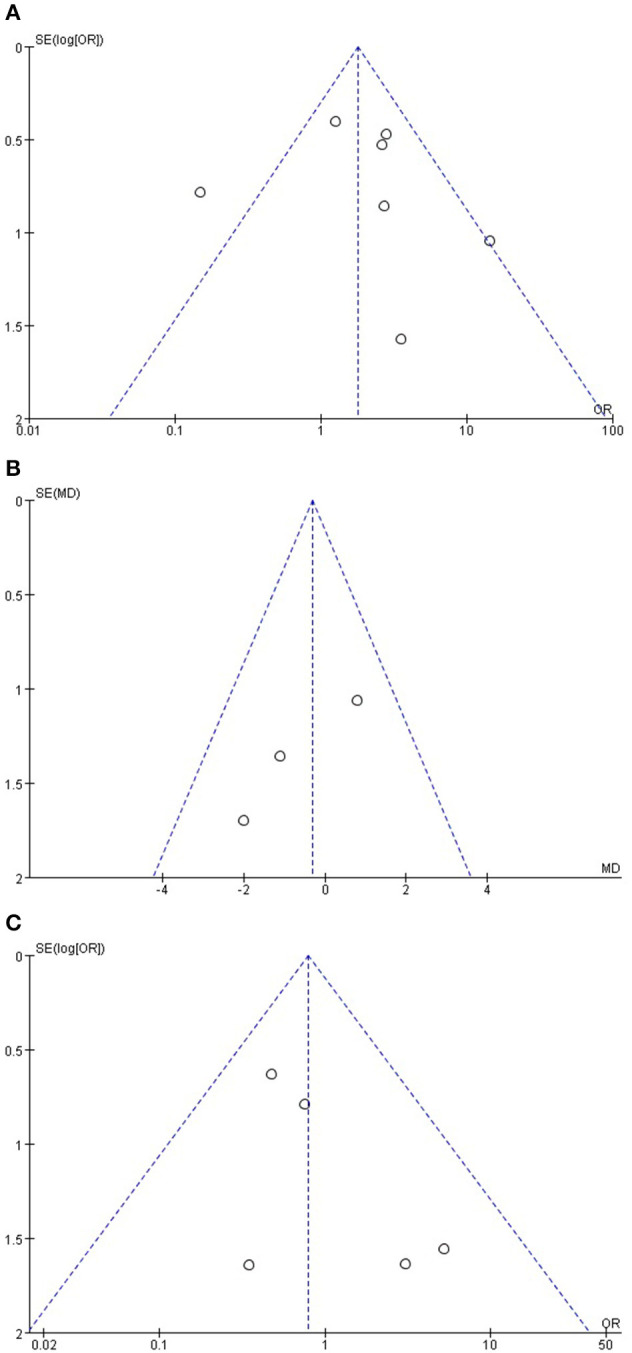
**(A)** Funnel plot based on the incidence of POPF, **(B)** length of hospital stay, and **(C)** mortality rate.

A funnel plot based on the incidence of POPF, length of hospital stay, and mortality rate is presented in Figure 6. The funnel plot does not show obvious asymmetry, and only one study lay outside the limits of the 95% CI for the incidence rate of POPF. No study lay outside the limits for mortality and length of hospital stay, indicating no obvious publication bias.

## Discussion

Although several studies have compared duct-to-mucosa and invagination anastomosis, increasing conflicting outcomes have been obtained. This is the first study to prove the statistically significant superiority of invagination over duct-to-mucosa anastomosis in terms of the rate of clinically relevant POPF after PD. The use of different definitions of POPF and the lack of a standardized surgical techniques make it difficult to compare results from different studies. In this article, a pancreatic fistula was defined using the newest version of the ISGPF criteria (2016), in which grades B and C POPF are termed clinically significant, while grade A is a biochemical fistula (7).

We found that in contrast to duct-to-mucosa anastomosis, invagination is easier to perform, and more of the pancreatic juice can be drained into the jejunum. Mechanically, when the duct-to-mucosa technique is applied, the main pancreatic duct is anastomosed, and other branch pancreatic ducts are closed by adhesion between the pancreatic stump and the jejunal or gastric wall. The technical requirements of duct-to-mucosa anastomosis are a dilated duct and a firm pancreas, and there are concerns about incomplete drainage of the pancreatic stump. The strengths of duct-to-mucosa technique include sufficient drainage of the main duct into the connected intestine as well as long-term preservation of duct patency. However, this technique does not allow drainage of the other branch ducts on the cut surface of the stump and requires a longer operative time. In patients with small pancreatic ducts, the operation is technically difficult and prolonged, and the risk of pancreatic injury and complications such as POPF are increased. Berger et al. (8) released a significantly higher rate of POPF after the duct-to-mucosa technique than the invagination technique and surprisingly found that the duct-to-mucosa technique was an independent risk factor of POPF. Therefore, the main advantage of duct-to-mucosa anastomosis is that it is suitable for patients with a small intestinal lumen, thicker pancreatic stump, or a hard pancreas. However, the disadvantage is that the method only facilitates drainage of the main pancreatic duct; it ignores the branch ducts. If the stump is not sutured well, a severe POPF may develop because of the presence of the branch pancreatic duct. A narrow main pancreatic duct makes this technique more difficult. In addition, the duct-to-mucosa technique is slightly complicated, involving many sutures and anastomoses with a high risk of needle-induced leakage.

The present study showed that invagination anastomosis is significantly superior in terms of the rate of clinically relevant POPF after PD. The mechanism underlying this superiority may be as follows; first, the operation time is much longer for duct-to-mucosa anastomosis than invagination because the former is a more complicated operation with a high number of anastomoses, making POPF more likely to develop; second, invagination involves the creation of a larger anastomosis to wrap the pancreatic duct for prevention of POPF, unlike the duct-to-mucosa technique which does not.

Patients with an exceedingly narrow main pancreatic duct, which cannot be seen on the pancreatic cut surface, are more likely to be treated using the invagination technique. The invagination technique is capable of complete drainage of both the pancreatic duct and the stump. But the need to mobilize a long length of the pancreatic stump and the need to place circumferential sutures, culminate in impairment, and disruption of the stump's blood supply (17). Therefore, the main advantages of invagination anastomosis are that both the pancreatic duct and pancreatic stump are buried in the jejunum and the operation is relatively simple. It is suitable for patients with a narrow pancreatic duct and a soft pancreas. However, because the pancreatic stump is exposed to the intestinal lumen with long-term exposure to digestive juice, the pancreatic stump is prone to erode and bleed, and the pancreatic duct is easily obstructed. This is a common shortcoming of all invagination anastomosis procedures. Patients with an excessive pancreatic stump are not suitable for invagination, especially through laparoscopy. If the anastomosis is forcibly inserted, not only will ischemia of the pancreatic stump occur but the pancreas will tear because of excessive tension on the suture.

The occurrence of POPF depends on several factors, including the diameter of the pancreatic duct, pancreatic texture, and anastomotic technique (18). Among the various factors that can contribute to POPF, the texture of the pancreas was clearly a major contributing factor in the present study. We found that patients with a soft pancreas were more likely to develop POPF after duct-to-mucosa than after invagination anastomosis. This is because it is difficult to anastomose a soft pancreas using the duct-to-mucosa technique. The diameter of the pancreatic duct is another important factor that can lead to POPF. We aimed to conduct a subgroup analysis of different diameters of the pancreatic duct, but only four articles included relevant data, and only two of these four articles had comparable data.

In the duct-to-mucosa technique, the size of the anastomosis is small with relatively less leakage than in the invagination technique, and tissue corrosion caused by the pancreatic fistula is limited. In contrast, invagination anastomosis involves a double-layer suture technique, which provides enough space for the accumulation of pancreatic juice. In addition, the anastomosis is relatively large, usually about 3 cm. Once POPF occurs, the amount of leakage is relatively large with a higher risk of infection. Each anastomosis has its own strengths and weaknesses.

The present meta-analysis showed no significant difference in the mortality rate or length of hospital stay between the two anastomosis techniques. The reason may be that invagination reduces the POPF rate but cannot reduce the overall complication rate. We searched relevant studies from all databases and found that all relevant RCTs revealed no significant difference between invagination and duct-to-mucosa anastomosis (10, 13, 14, 19, 20), which can support the hypothesis that invagination reduces the POPF rate but cannot reduce the overall complication rate. Additional studies are needed to define the optimal technique of pancreatic reconstruction after PD. Such studies could provide a stimulus for future prospective randomized trials focusing on variations in technique and interventions in pancreatic surgery.

Our study has several strengths. We included high-quality studies, and all of them were RCTs. In addition, we used the newest standard definition of POPF (ISGPS 2016). Moreover, we identified a new method with which to reduce the rate of clinically relevant POPF without increasing the mortality rate or length of hospital stay.

Our study also has several limitations. First, some studies reported in languages other than English and, possibly, some unpublished studies were excluded; this may have contributed to selection bias. Second, several known risk factors for POPF, such as the diameter of the main pancreatic duct, stent use, and octreotide use, could not be explored in the subgroup analysis because of the lack of comparable information in the included trials. Third, indication for PD may be a potential bias due to heterogenicity. Fourth, although we believe that the conclusion of this meta-analysis is the most dependable conclusion obtained in this field to date, it might not be the final conclusion. Future studies may change the conclusion, although the probability seems low.

## Conclusion

This study demonstrated superiority of invagination anastomosis over duct-to-mucosa anastomosis in reducing the risk of a Grade B/C postoperative pancreatic fistula using the ISGPS 2016 definition. The effect of invagination in reducing pancreatic fistula formation is obvious in patients with a soft pancreas. We found a lower rate of clinically relevant postoperative pancreatic fistula in the invagination group, in patients with a soft pancreas.

## Data Availability Statement

The original contributions presented in the study are included in the article/supplementary material, further inquiries can be directed to the corresponding author/s.

## Author Contributions

WL and ZC: study design and data interpretation. WL: literature search, data retrieval, data analysis, and writing of the manuscript. WL, ZC, and TZ: critical revision and final approval. All authors contributed to the article and approved the submitted version.

## Conflict of Interest

The authors declare that the research was conducted in the absence of any commercial or financial relationships that could be construed as a potential conflict of interest.

## Abbreviations

PD: Pancreaticoduodenectomy
POPF: Postoperative pancreatic fistula
FRS: fistula risk score
ISGPS: International Study Group of Pancreatic Surgery
CIs: Confidence intervals.
